# Effect of neoadjuvant chemotherapy on the expression of hormone receptors and Ki67 in Chinese breast cancer patients: A retrospective study of 525 patients

**DOI:** 10.7555/JBR.32.20170059

**Published:** 2018-02-27

**Authors:** Yu-tuan Wu, Xin Li, Lin-jie Lu, Lu Gan, Wei Dai, Yan-ling Shi, Vishnu Prasad Adhikari, Kai-nan Wu, Ling-quan Kong

**Affiliations:** 1. Department of Endocrine and Breast Surgery, the First Affiliated Hospital of Chongqing Medical University, Chongqing 400016, China; 2. Department of Thyroid and Breast Surgery, Liuzhou People’s Hospital, Liuzhou, Guangxi 545006, China; 3. Department of Oncology and Radiology, the First Affiliated Hospital of Chongqing Medical University, Chongqing 400016, China.

**Keywords:** breast cancer, neoadjuvant chemotherapy, hormone receptors, Ki67

## Abstract

This study was designed to investigate the effect of neoadjuvant chemotherapy on the expression of hormone receptors and Ki67 in Chinese female breast cancer patients. The expression of estrogen receptor (ER), progesterone receptor (PR) and Ki67 among 525 neoadjuvant chemotherapy cases was studied by immunohistochemistry. Differences between specimens made through preoperative core needle biopsy and excised tissue biopsy were observed. The positive rates of ER, PR and Ki67 in core needle biopsy and excised tissue biopsy were 65.3% and 63.2%, 51.0% and 42.6%, 65.6% and 43.4%, respectively. The expression of ER, PR and Ki67 in core needle biopsy and excised tissue biopsy had no statistically significant difference. However, after neoadjuvant chemotherapy, the discordance rates of ER, PR and Ki67 were 15.2% (79/521), 26.9% (140/520) and 44.8% (225/502), respectively. The ER, PR and Ki67 status changed from positive to negative in 7.5% (39/521), 13.3% (69/520) and 21.1% (106/502) of the patients, whereas ER, PR and Ki67 status changed from negative to positive in 7.7% (40/521), 13.6% (71/520) and 23.7% (119/502) of the patients, respectively. These results showed that the status of some biomarkers changes after neoadjuvant chemotherapy and biomarker status needs to be reexamined to optimize adjuvant systemic therapy and better prognosis assessment.

## Introduction

Breast cancer is the most common and deadly cancer among females^[1]^. Neoadjuvant chemotherapy, the primary systemic treatment, has become a standard treatment to shrink the tumor and improve the chance of breast conserving surgery for operable breast cancer patients, because it can increase disease-free survival and overall survival rates compared with postsurgical adjuvant chemotherapy^[2^–^3]^. Neoadjuvant chemotherapy is completed before surgery, except for patients who have disease progression during treatment, because the progression may put them at higher risk of surgery^[4]^. Meanwhile, hormone receptor (HR) assays have become a standard practice for clinicians in endocrine treatment^[5]^. The hormone-dependent nature of breast cancer is the basis for endocrine therapy that benefits patients with positive estrogen receptor (ER) and progesterone receptor (PR)^[6^–^7]^. Previous studies have reported that neoadjuvant chemotherapy affects biomarker status in breast cancer, so a question has been raised about how neoadjuvant chemotherapy modulates these markers. However, the results of several studies on the effect of neoadjuvant chemotherapy on biomarker status in breast cancer are conflicting^[8^–^10]^.

In this study, we retrospectively analyzed HR expression and Ki67 in specimens of core needle biopsy (CB) and excised tissue biopsy (EB) from 525 breast cancer patients who received neoadjuvant chemotherapy and evaluated the impact of neoadjuvant chemotherapy on the expression of these two biomarkers. We further determined whether these tumor specimens should be reexamined after neoadjuvant chemotherapy.

## Patients and methods

### Patients

**Fig.1:**
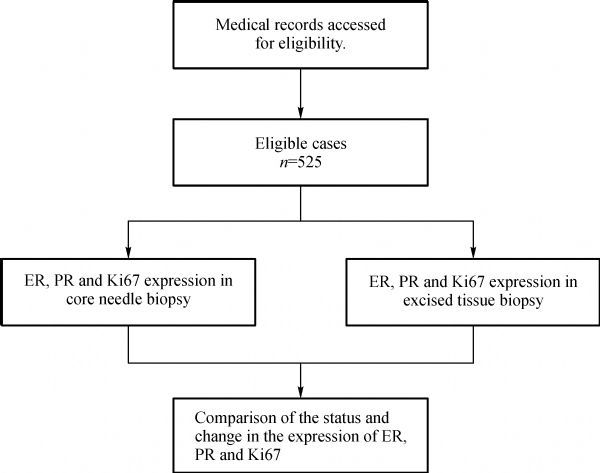
The flow diagram of case selection.

We retrospectively collected data of patients with invasive breast cancer who had undergone preoperative biopsy and subsequent surgical resection after neoadjuvant chemotherapy at the First Affiliated Hospital of Chongqing Medical University (Breast Cancer Center of Chongqing, China). We reviewed pathology reports of breast cancer patients from April 2012 to December 2015 through the hospital medical record system, and extracted pertinent data. Eligible participants were breast cancer patients who had received neoadjuvant chemotherapy and had both core needle biopsy and excised tissue biopsy. After screening the cases with reliable history and unified pathology reports, we selected 525 neoadjuvant chemotherapy cases (***Fig. 1***). As for core needle biopsy, considering the heterogeneous nature of the tumor, four to six needles were punctured in the tumor as evenly as possible in each specimen. After neoadjuvant chemotherapy, the patients underwent tumor resection. The needle and excised biopsy specimens of these cases were preserved following the standards, and immediately transferred for pathology and immunohistochemistry (IHC) detection at the Clinical Pathology Diagnostic Center of Chongqing, Chongqing Medical University.

This study was approved by the Administration Ethics Committee of the First Affiliated Hospital of Chongqing Medical University and conducted in accordance with the Principles of Helsinki Declaration and patient consent was not required because of the retrospective nature of the study. 

### Immunohistochemistry

We evaluated the difference in concordance rates of ER, PR and Ki67 expression between the preoperative biopsy and resected tumor specimens after neoadjuvant therapy by immunohistochemistry. All specimens were fixed in 10% neutral buffered formalin. Specimens of primary biopsy were fixed for a minimum of 6 hours before processing, while surgical specimens were fixed within 1 hour after resection for at least 8 hours. The status of ER, PR and Ki67 was collected from pathology reports, and the information on adjuvant therapies was obtained from the Breast Cancer Registry of Breast Cancer Center, the First Affiliated Hospital of Chongqing Medical University. The status of ER, PR and Ki67 was assessed by monoclonal antibody-based immunohistochemistry (BenchMark automated system; Ventana Medical Systems, Tucson, AZ; with commercially available antibodies). All these were performed following the standard procedure at the Clinical Pathology Diagnosis Center of Chongqing Medical University. 

The Allred score is an indicator of HR status by immunohistochemistry to evaluate the potential magnitude of HR change^[10]^. The calculation was done in two steps. First, a proportional score, representing the proportion of positive-staining tumor cells (0, no stained cells; 1, 0<stained cells<1%; 2, 1%≤stained cells<10%; 3, 10%≤stained cells<33%; 4, 33%≤stained cells<66%; 5, 66%≤stained cells), was determined. Second, a stage score, representing the average intensity stage of positive tumor cells (0, none; 1, weak intensity; 2, intermediate; 3, strong), was determined. The Allred score is obtained by adding the proportional score to the stage score. An Allred score>2 was used to define tumor HR positivity. Cancer cell proliferation was assessed by identification of Ki67-positive cells within the tissue section. The proliferation rate is shown as the number of Ki67-positive cells in 100 carcinoma cells per section. In accordance with the study by Fasching *et al.*, a proportion of ≥13% positively stained cells, the Ki67 index to distinguish luminal B from luminal A tumors, was used as the cut-off point for Ki67 status^[11^–^12]^.

### Statistical analyses

The association of biomarkers between the CB specimens and the EB specimens were analyzed using the McNemar test^[13]^. For statistical procedures, Microsoft Excel 2010 and SPSS 22.0 software were used. All statistical tests were two-sided and *P*<0.05 was considered statistically significant.

## Results

### Patient demographic and baseline characteristics

The demographic and clinicopathologic characteristics of the participants are summarized in ***Table 1***. The mean age was 49±9 years (34.9% were 45 years or younger), and approximately half (50.9%) of the patients were premenopausal. Pathological tumor stage, nodal stage, clinical stage, histologic types and histologic grade were predominantly pT1 (61.7%), pN0 (36.4%), stage II (76.2%), ductal type (94.3%), and grade 2 (74.7%), respectively. All patients used anthracyclines or taxanes based regimens for neoadjuvant chemotherapy. Mastectomy was mostly performed after neoadjuvant chemotherapy.

**Tab.1:** Baseline and clinicopathologic characteristics of the patients

Characteristics	Cases (*n*=525)	
	*n*	%
Age, years		
≤45	183	34.9
>45	342	65.1
Menopausal status		
Premenopausal	267	50.9
Postmenopausal	258	49.1
Pathological tumor stage		
pT1	324	61.7
pT2	169	32.2
pT3	32	6.1
pT4	0	0
Pathological nodal stage		
pN0	191	36.4
pN1	108	20.6
pN2	78	14.8
pN3	148	28.2
Clinical stage		
I	85	16.2
II	400	76.2
III	40	7.6
IV	0	0
Histological type		
Ductal	495	94.3
Lobular	5	0.9
Mixed type	21	4.0
Unknown	4	0.8
Histological grade		
G1	8	1.5
G2	392	74.7
G3	59	11.2
Unknown	66	12.6
Surgery		
Conserved breast	8	1.5
Mastectomy	517	98.5

### Expression of hormone receptors and Ki67

The expression of hormone receptors and Ki67 in breast cancer tissues are summarized in ***Table 2 ***and*** 3***. The positive rates of ER, PR and Ki67 in CB and EB were 65.3% and 63.2%, 51.0% and 42.6%, 65.6% and 43.4%, respectively. The expression of ER, PR and Ki67 between CB and EB was found to have no statistically significant difference (*P*>0.05).

**Tab.2:** Biomarker expression of ER, PR and Ki67 in CB and EB specimens in neoadjuvant chemotherapy (NAC) cases[n/N(%)]

BiomarkersBiomarker status		Cases (*n*=525)		
		CB	EB	McNemar test
ER	Negative	182/525 (34.7)	193/525 (36.8)	*P*>0.05
	Positive	343/525 (65.3)	332/525 (63.2)	
PR	Negative	256/522 (49.0)	300/523 (57.4)	*P*>0.05
	Positive	266/522 (51.0)	223/523 (42.6)	
Ki67	Negative	173/503 (34.4)	295/521 (56.6)	*P*>0.05
	Positive	330/503 (65.6)	226/521 (43.4)	

**Tab.3:** Change of ER, PR and Ki67 status in CB and EB specimens in paired neoadjuvant chemotherapy (NAC) cases[n/N(%)]

Biomarkers	Change	Cases (*n*=525)^a^	Total change (%)
ER	No change	442/521 (84.8)	79/521 (15.2)
	Neg→Pos	40/521 (7.7)	
	Pos→Neg	39/521 (7.5)	
PR	No change	380/520 (73.1)	140/520 (26.9)
	Neg→Pos	71/520 (13.6)	
	Pos→Neg	69/520 (13.3)	
Ki67	No change	277/502 (55.2)	225/502 (44.8)
	Neg→Pos	119/502 (23.7)	
	Pos→Neg	106/502 (21.1)	

Note: Pos→Neg: positive to negative; Neg→Pos: negative to positive.

***Table 3*** presents changes in the status of ER, PR and Ki67 in CB and EB cases in detail. Comparing the concordance rates of the CB and EB groups, ER was 84.8% (442/521), PR was 73.1% (380/520) and Ki67 was 55.2% (277/502). The discordance rates of ER, PR and Ki67 were 15.2% (79/521), 26.9% (140/520) and 44.8% (225/502). Meanwhile, 7.7% (40/521) cases and 13.6% (71/520) cases had ER and PR positive conversion, respectively. In 21.1% (106/502) cases, Ki67 status changed from positive to negative after neoadjuvant chemotherapy, while in 23.7% (119/502) cases, Ki67 status changed from negative to positive.

## Discussion

A number of studies have revealed concordance between HR and Ki67 after neoadjuvant chemotherapy^[8]^. Discordance of the hormone receptor status ranged from 8% to 33% in breast cancer patients who received neoadjuvant chemotherapy. About half of the studies reported a discordance rate of 2.5%–17.0% in ER receptor status and a discordance rate of 5.9%–51.7% in PR, respectively. Several studies have been done to evaluate the effect of neoadjuvant chemotherapy on biomarker status in breast cancer with conflicting results^[9^,^14]^. Because these studies were conducted using a limited number of specimens, there might not be sufficient evidence to detect significant differences. For instance, Arens *et al*. analyzed biomarkers of CB and EB in 25 patients receiving neoadjuvant chemotherapy and 30 patients without neoadjuvant chemotherapy, and concluded that neoadjuvant chemotherapy had no effect on the expression of biomarkers^[9]^. By contrast, our study, with 525 neoadjuvant chemotherapy cases, is more likely to detect small but statistically significant differences.

Appropriate systemic treatment of breast cancer requires the knowledge of HR status, especially after neoadjuvant chemotherapy. In our study, the differences of ER, PR and Ki67 expression between CB and EB were not statistically significant. However, there is a certain discordance rate of ER, PR and Ki67 expression between CB and EB, which is in accordance with the results of some previous studies. These changes verify the presumption that the shift in biomarkers is elicited by neoadjuvant chemotherapy and random changes induced by heterogeneity, laboratory procedures and observer variability. Due to improvement in sampling and detecting techniques, tumor heterogeneity would be a negligible factor of biomarker shift. However, the effect of neoadjuvant chemotherapy on biomarker expression changes could hardly be ignored.

After neoadjuvant chemotherapy, 13.6% (71/520) initially PR negative cases became positive. As for ER expression, 7.7% (40/521) negative cases transformed to positive ones after neoadjuvant treatment. As previously reported, possible mechanisms for HR expression shift in breast cancer cells caused by chemotherapy were as follows: First, chemotherapy induced the regression to a positive hormone receptor status, since all cells originally derived from well-differentiated hormone receptor positive breast cancer cells^[8]^; Another explanation would be selection of tumor cells clone during treatment, with selective disappearance of either HR-positive or HR-negative tumor cells. It is generally known that HR-negative tumors are more sensitive to chemotherapy than HR-positive ones^[10]^. Chemotherapy could up-regulate some proteins favoring the expression or re-expression of HR in the tumor nuclei. The theory that HR-negative tumors are more sensitive to chemotherapy than HR-positive tumors is explained by up-regulation of HR. Although ER expression shift did not show significant difference, 7.5% positive cases became negative after neoadjuvant chemotherapy. A novel theory called neoendocrino chemotherapy can better explain these results^[15]^. This novel theory focuses on enhancing tumor cell sensitivity to chemotherapy by providing endocrine hormones to patients before and/or during chemotherapy, and it may offer a new therapy for breast cancer. That is to say, some HR positive tumor cells may be more sensitive to chemotherapy. This novel theory is also concordant with the results from Kitagawa *et al*., indicating that negative conversion of HR positive cases were more frequently observed in patients under 50 years (the average menopausal age of natives), since their hormone level is higher than those over 50 years^[16]^.

Tacca *et al*. found patients with HR negative tumors which switched to a positive status after neoadjuvant chemotherapy had better overall survival and disease-free survival than patients whose tumors remained HR negative^[10]^. Hirata *et al*. verified patients whose HR status shifted from negative to positive after neoadjuvant chemotherapy, if administered endocrine treatment, had a better prognosis than patients who were HR-negative both before and after neoadjuvant chemotherapy^[17]^; but for those who did not receive endocrine treatment, the prognosis was worse. These results indicate that it is necessary to assess lesion HR status both before and after neoadjuvant chemotherapy, and endocrine treatment is also essential for patients with positive HR status conversion. The poor prognosis for patients with positive HR conversion and without adjuvant endocrine treatment indicates the necessity to evaluate the biopsy specimens both before and after neoadjuvant chemotherapy; the pre- and post-neoadjuvant chemotherapy HR status would help determine the application of adjuvant endocrine treatment in patients. Endocrine treatment can be applied in patients with HR positive tumor at least once, that is, either before or after neoadjuvant chemotherapy.

In most of the previously reported studies, a decrease in Ki67-associated proliferation was observed, which is in accordance with our study^[9]^. In the neoadjuvant chemotherapy cases, there are 34.4% negative cases and 65.6% positive cases in CB specimens, while 56.6% negative cases and 43.4% positive cases in EB specimens after neoadjuvant chemotherapy. In addition, 21.1% (106/502) cases have negative conversion, which could be attributed to the disturbance of signal transduction pathways caused by chemotherapeutic agents^[9]^. Meanwhile, 23.7% (119/502) cases have positive conversion, possibly due to the fact that some tumor cells were resistant to the chemotherapeutic agent. A recent research indicates that post-neoadjuvant chemotherapy Ki67 levels provided more prognostic information than pre-neoadjuvant chemotherapy ones^[18]^. Patients with a high level of post-treatment Ki67 had higher risks for relapse and death compared with those who had a low or intermediate level of Ki67^[19]^. The Ki67 index is a predictive marker for pathologic complete response. Patients with a low level of Ki67 showed a comparable outcome with patients with a pathologic complete response^[19^–^20]^. The 2011 and 2013 St. Gallen Consensus Conference added Ki67 as a proliferation marker for breast cancer subtypes like luminal A, luminal B, as well as triple negative basal-like and HER2 overexpressing types^[21^–^22]^. These breast cancer molecular subtypes have been proposed as risk factors and prognosis indicators^[23]^. For the above reasons, it is necessary to reexamine Ki67 in patients with residual disease after neoadjuvant chemotherapy for better subsequent therapy planning and prognosis assessment.

The limitations of our study are as follows: (1) Our study is retrospective, so biomarker information was not acquired from patients who received neoadjuvant chemotherapy. (2) We did not stratify the neoadjuvant chemotherapy patients based on their chemotherapy regimens. (3) Patients’ prognostic information was not available, so the impact of biomarker conversion on prognosis was not evaluated. Further research should be conducted prospectively on change of biomarker expression, and the prognosis of breast cancer patients after neoadjuvant chemotherapy should be assessed. Regardless of the limitations, our study has shown that neoadjuvant chemotherapy could modulate tumor biomarker status, indicating the significance of evaluating biomarker status both before and after neoadjuvant chemotherapy.

In summary, in our study, the discordance rates of ER, PR and Ki67 were 15.2%, 26.9%, 44.8% before and after neoadjuvant chemotherapy, respectively. Meanwhile 7.7% and 13.6% cases showed positive conversion of ER and PR expression, respectively, after neoadjuvant chemotherapy. Regardless of the reasons for ER, PR and Ki67 status switch, changes of ER, PR and Ki67 expression should not be ignored by oncologists. Re-examination of the biomarkers should be conducted in certain situations to optimize the adjuvant systemic therapy and assess patient’s prognosis.
